# Defective Differentiation of Myeloid and Plasmacytoid Dendritic Cells in Advanced Cancer Patients is not Normalized by Tyrosine Kinase Inhibition of the Vascular Endothelial Growth Factor Receptor

**DOI:** 10.1155/2007/17315

**Published:** 2007-12-13

**Authors:** H. van Cruijsen, K. Hoekman, A. G. M. Stam, A. J. M. van den Eertwegh, B. C. Kuenen, R. J. Scheper, G. Giaccone, T. D. de Gruijl

**Affiliations:** ^1^Department of Medical Oncology, VU University Medical Center, De Boelelaan 1117, 1081 HV Amsterdam, The Netherlands; ^2^Department of Pathology, VU University Medical Center, De Boelelaan 1117, 1081 HV Amsterdam, The Netherlands

## Abstract

Tumor-derived vascular endothelial growth factor (VEGF) has previously been identified as a causative factor in the disturbed differentiation of myeloid dendritic cells (DC) in advanced cancer patients. Here, we investigated the potential of vascular endothelial growth factor receptor (VEGFR) tyrosine kinase (TK) inhibition to overcome this defective DC differentiation. To this end, peripheral blood DC (PBDC) precursor and subset frequencies were measured in 13 patients with advanced cancer before and after treatment with AZD2171, a TK inhibitor (TKI) of VEGFR, coadministered with gefitinib, and an epidermal growth factor receptor (EGFR) TKI. Of note, not only myeloid DC but also plasmacytoid DC frequencies were significantly reduced in the blood of the cancer patients prior to treatment, as compared to healthy controls. Moreover, besides an accumulated population of immature myeloid cells (ImC), a population of myeloid suppressor cells (MSC) was significantly increased. Upon systemic VEGFR TK inhibition, DC frequencies did not increase, whereas the rate of circulating MSC showed a slight, but not significant, decrease. In conclusion, TK inhibition of VEGFR with AZD2171 does not restore the defective PBDC differentiation observed in advanced cancer patients.

## 1. INTRODUCTION

Defective dendritic cell (DC) differentiation, maturation, and functionality are possible mechanisms underlying impaired antitumor immunity in cancer patients [1]. DCs play a central role in the immune system as powerful antigen-presenting cells, and are essential for the induction of tumor-specific T-cell-mediated immune responses [2]. In cancer patients, the frequencies of circulating DCs are significantly lower as compared to healthy individuals [3–5]. Accumulation of immature myeloid cells (ImC) and functionally impaired DCs has been documented in blood, tumors, and tumor-draining lymph nodes and found to be a poor prognostic factor [3, 4, 6]. Preclinical studies show that tumor-induced inhibition of DC differentiation is mediated by tumor-derived soluble factors such as IL-10, IL-6, M-CSF, prostaglandins, and vascular endothelial growth factor (VEGF) [7–11].

VEGF, produced by most tumors, is a strong inhibitor of myeloid DC differentiation in vitro [7] and affects the early stages of functional DC differentiation [12, 13]. High-systemic VEGF levels, present in most cancer patients, correlate with low DC frequencies [3, 4], while abnormally elevated numbers of immature DC precursors reportedly decreased in three out of three cancer patients during treatment with the anti-VEGF antibody bevacizumab [3].

VEGF is also one of the most important proangiogenic molecules and induces proliferation, differentiation, and migration of endothelial cells in tumors. Over the past decades, many trials with inhibitors of angiogenesis have been conducted and have resulted in the registration of bevacizumab as an anticancer therapy [14]. VEGF exerts its effect via binding to three tyrosine kinase (TK) receptors, VEGFR-1, -2, and -3, which are mainly, but not exclusively, present on endothelial cells (VEGFR-1, and -2) and lymphatic endothelium (VEGFR-3) [15]. Blocking VEGF signaling by inhibiting TK activity of its receptor, is a promising anticancer strategy. AZD2171 is a novel potent inhibitor of VEGFR-2 kinase activity, with additional activity against VEGFR-1 and -3 [16].

Currently, AZD2171 is being evaluated in clinical trials as an oral anticancer agent with antiangiogenic effects in a variety of solid tumors. To further investigate the relationship between VEGFR signaling and DC differentiation, we evaluated the effect of administration of the VEGFR inhibitor AZD2171 on peripheral blood DC (PBDC) subsets in advanced cancer patients. To our knowledge, this is the first study to monitor DC subsets in the blood of cancer patients who are treated with a VEGFR tyrosine kinase inhibitor (TKI).

## 2. MATERIAL AND METHODS

### 2.1. Patients and healthy donors

Between May 2004 and December 2004, 13 patients of the VU Medical Center, Amsterdam, were selected to participate in a phase I study combining AZD2171 (AstraZeneca, Wilmington, DE, USA) a VEGFR TKI [16], with gefitinib (AstraZeneca, Wilmington DE), a TKI of the epidermal growth factor receptor (EGFR). The dose of AZD2171 was escalated in small, consecutive cohorts of advanced cancer patients coadministered with 250 mg gefitinib in order to establish a maximum-tolerated dose. Thirteen patients (three women and ten men) were included in one of the three-AZD2171 dosing cohorts: 20 mg (n = 3), 30 mg (n = 7), and 45 mg (n = 3). Major inclusion criteria were locally advanced or disseminated disease, which was refractory to standard therapy, age over 18 years, and a performance status of 0–2. Excluded were patients with impaired renal or liver function or inadequate bone marrow reserve. The clinical trial was approved by the Medical Ethical Committee, and after obtaining informed consent, blood from the patients (mean age of 52, range from 31 to 66) was drawn before treatment and after four or five weeks of daily oral dosing of both drugs (depending on the dosing schedule of the protocol). A variety of primary tumors was represented: colon cancer (n = 3), mesothelioma (n = 2), melanoma (n = 2), fibrosarcoma, osteosarcoma, renal cell cancer, cervical cancer, pancreas cancer, and NSCLC (all n = 1). After four or five weeks of treatment, tumor status was evaluated according to RECIST [17].

Two control groups were included. First, blood was drawn from nine age- and sex-matched healthy donors (two women and seven men, mean age of 47, range from 32 to 55) to collect peripheral blood mononuclear cells (PBMCs). A second control group consisted of four advanced non-small cell lung cancer (NSCLC) patients (a woman and three men, mean age of 61, range from 51 to 66) who received gefitinib monotherapy. PBMCs were obtained at baseline and four weeks after daily dosing of 250 mg gefitinib.

### 2.2. PBDC monitoring

PBMCs were isolated by Ficoll density-gradient centrifugation (Lymphoprep, Oslo, Norway) within 24 hours of blood sampling. FACS analysis (Becton Dickinson, Franklin Lakes, NJ, USA) was performed to measure peripheral blood DC (PBDC) subset frequencies and their maturation status using four-color staining with antibodies directly conjugated with fluorochromes FITC, PE, PerCP-Cy5, or APC. Monoclonal antibodies against the following markers were used: CD3, CD11c, CD14, CD19, CD56, CD86, CD123, EGFR, HLA-DR (all BD Biosciences, San Jose, CA, USA), BDCA-1, BDCA-2, BDCA-3, BDCA-4 (all Miltenyi Biotec GmbH, Bergisch Gladbach, Germany), VEGFR-1, VEGFR-2 (R&D Systems, Minneapolis, MN, USA), and CD33 (Immunotech, Marseille, France). A gate was set on the population containing lymphocyte and monocytes, based on the forward and sideward scatter plots to avoid erythrocytes, cell debris, and neutrophil contamination.

Data were obtained from a minimum of 150.000 cells and were analyzed using CellQuest software (Macintosh). Results are shown as percentages of the total number of PBMCs. Labeled isotype-matched IgG antibodies were used to determine background fluorescence in each analysis. Median fluorescence indices were calculated by dividing the median expression of the antibody of interest by the median background fluorescence as determined by the isotype-matched IgG antibody.

**Figure 1:**
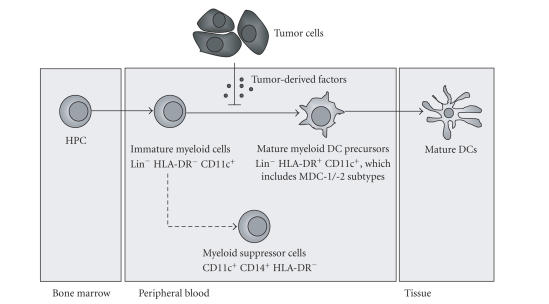
A model of myeloid dendritic cell differentiation under cancer conditions, in which tumor-derived factors exert their inhibitory effect at the stage of immature Lin^−^CD11c^+^ HLA-DR^−^ myeloid cells (ImCs), blocking their differentiation into mature myeloid DC precursors (pMDC), while, simultaneously, skewing their differentiation towards a population of CD14^+^ HLA-DR^neg/low^ myeloid suppressive cells (MSCs). HPCs: hematopoietic cells; Lin: lineage markers (CD3, CD14, CD19, and CD56); MDC: myeloid dendritic cell.

Different PBDC precursor populations and subsets were studied, reflecting different stages of development (schematically presented for the myeloid lineage in Figure 1).

**Myeloid DC (MDC)**

Immature myeloid cells (ImC), previously identified as MDC or macrophage precursors in varying stages of differentiation [18], were defined as positive for CD11c, but negative for the lineage (Lin) markers CD3 (T cells), CD14 (monocytes), CD19 (B cells), and CD56 (NK cells), as well as for HLA-DR as previously described [3]. More mature MDC precursors (pMDC) were defined as CD11c^hi^ Lin^−^HLA-DR^+^, and the frequencies of two MDC subsets contained within this population were more specifically determined based on the expression of blood DC antigen (BDCA) markers: DCs belonging to the so-called myeloid DC subset 1 (MDC-1) were identified as CD11c^hi^, ${\text{CD14}}^{-}$, and BDCA-1/CD1c^+^, and MDC-2 were detected as CD11c^+^, ${\text{CD14}}^{-}$, and BDCA-3/CD141^+^[19].

**Plasmacytoid DC (PDC)**

PDCs were detected as ${\text{CD11c}}^{-}$, ${\text{CD14}}^{-}$, CD123^hi^, and BDCA-2^+^, or as Lin^−^BDCA4^+^. As previously described, in blood, both BDCA-2 and BDCA-4 are exclusively expressed on PDCs [19].

### 2.3. Measurement of circulating VEGF

At the time of PBMC isolation, serum samples were also collected. After clot formation (60 minutes at room temperature) and centrifugation, serum was harvested and stored at −80°C. Circulating VEGF levels were measured in serum samples with a Quantikine ELISA kit (R&D systems, Minneapolis, MN, USA), following the manufacturer's instructions.

### 2.4. Statistical analysis

Since we could not assume a normal distribution of the DC subset frequencies, we applied nonparametric tests. Percentages of PBDCs and levels of circulating VEGF obtained from samples during VEGFR TK inhibition were compared with baseline values using a Wilcoxon signed ranks test and a paired t test, respectively, to determine statistical significance. In addition, the Mann-Whitney U test was used to determine significance of differences between patient and healthy donor data. Differences were considered to be statistically significant when *P* < .05.

## 3. RESULTS

Thirteen patients included in the phase I study combining AZD2171 with gefitinib participated in the DC monitoring study. Preliminary efficacy of treatment was observed in four patients. In one patient, a partial remission was observed; in three patients a decrease in tumor size was noticed, but this decrease did not reach the criteria for partial remission and was considered a stable disease.

### 3.1. VEGFR-1, VEGFR-2, and EGFR expression on the studied PBDC subsets

Different PBDC precursor populations and subsets were studied, reflecting different stages of development, as described in “Materials and Methods” section (see also Figure 1). To establish the ImC and pMDC and the MDC and PDC subsets as viable targets for the employed VEGFR and EGFR TKIs, expression levels of VEGFR-1, VEGFR-2, and EGFR were determined and expressed by their median fluorescence indices (med FI). Both VEGFR-1 and VEGFR-2 were expressed, albeit at generally low levels, on ImC and on pMDC (med FI range from 2.1 to 3.1), as well as on the MDC-1, MDC-2, and PDC subsets (med FI range from 2.0 to 3.8). In contrast, EGFR was not expressed on any of the studied PBDC subsets or precursor stages (med FI range from 0.4 to 0.9).

### 3.2. PBDC frequencies before and after VEGFR TK inhibition

#### 3.2.1. Immature myeloid cells (ImC) and MDC precursors (pMDC)

**Figure 2:**
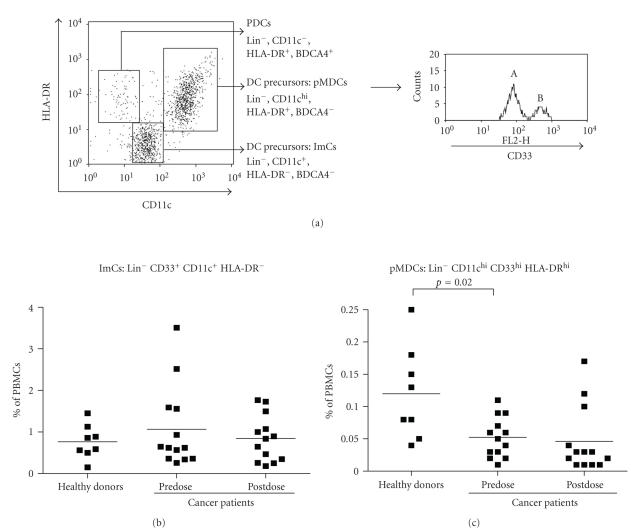
Typical results of blood DC analysis based on the absence of lineage (Lin) markers (CD3, CD14, CD19, and CD56) in a patient with mesothelioma. Events are gated for the absence of Lin marker expression and the presence of CD33 expression. Lin^−^ cells, that is, immature myeloid cells (ImCs) and more mature MDC precursors (pMDC), were distinguished from plasmacytoid dendritic cells (PDC) by BDCA-4 expression (a). CD33 expression was determined for the CD11c^hi^ HLA-DR^+^ population. Within the HLA-DR positive population (pMDC), two subpopulations (A and B) were discernable based on intermediate and high CD33 expression (a). Results for immature myeloid cells (ImC and Lin^−^ HLA-DR^−^) (b) and mature MDC precursors (pMDC and Lin^−^ CD33^hi^ HLA-DR^hi^) (c) are shown for healthy donors and for cancer patients before (predose) and after VEGFR inhibition (postdose) (c). Peripheral blood DC percentages are of total peripheral blood mononuclear cells. In both graphs, individual values and the means are shown.

Almand et al. [3]reported an accumulation of immature Lin^−^HLA-DR^−^ ImC in the blood of cancer patients, while more mature Lin^−^HLA-DR^+^MDC precursors (pMDC) were found to be reduced. To further characterize these populations, we included the myeloid lineage-associated markers CD11c and CD33. BDCA-4 (neuropilin-1, expressed by PDC) was included in our analyses to ascertain if the previously reported accumulation of immature DC precursors contained within the Lin^−^DR^−^ImC fraction might actually involve PDCs. Typical results, shown in Figure 2(a), demonstrate that the Lin^−^HLA-DR^−^ ImC were CD11c^+^ and did not express BDCA-4, and were, therefore, unlikely to include PDCs. In Figure 2(a), CD33 expression is shown for the Lin^−^CD11c^hi^ HLA-DR^+^ pMDC population. Within this pMDC population, two subpopulations were clearly discernable based on different CD33 expression levels (indicated as A and B in Figure 2(a)). As CD33 is a myeloid marker known to be associated with PBDC differentiation and previously shown to be highly expressed on the MDC-1 and MDC-2 subsets but not on an immature DC progenitor subset [20], we take this CD33^hi^ population to represent a more mature MDC population.

We did observe a slight accumulation of ImC in the advanced cancer patients (based on pretreatment frequencies), but this did not reach the level of significance when compared to healthy donors (Figure 2(b)). Of note, within the pMDC population, cells with high CD33 expression levels (population B in Figure 2(a)) were significantly decreased in the cancer patients before treatment, as compared to the healthy donors (***P*** = .02, Figure 2(c)). In the cancer patients, VEGFR TK inhibition through AZD2171 treatment did not significantly change the frequencies of the MDC precursors in any of these different stages of development (Figures 2(b), 2(c)). Of note, Figure 2(c) shows three outlying postdose pMDC frequencies well above the mean of the postdose cancer patient group. In conjunction with a restoration of pMDC frequencies to values within the range observed for healthy donors, these three patients experienced clinical benefit from AZD2171 and gefitinib treatment: two patients had a minor response and the third patient had stable disease lasting for 31 weeks.

#### 3.2.2. Myeloid suppressor cells (MSC)

**Figure 3:**
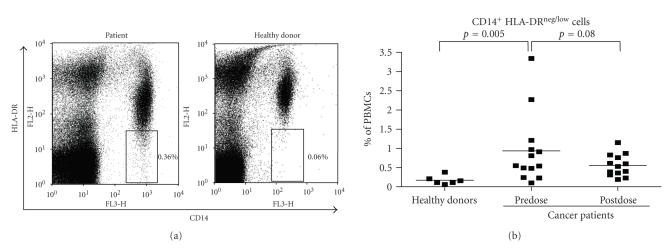
Results for myeloid suppressor cells (CD14^+^ HLA-DR^neg/low^) in healthy donors and in cancer patients before and after VEGFR inhibition. Typical results of myeloid suppressor cells in a patient and one healthy donor are shown (a). Peripheral blood DC percentages are of total peripheral blood mononuclear cells. The individual values and the means for healthy donors and cancer patients before (predose) and after VEGFR TKI inhibition (postdose) are shown (b).

We identified a population of CD14^+^HLA-DR^neg/low^ cells in the blood of our patients (Figure 3(a)), which, before treatment, was significantly increased as compared to healthy donors (***P*** = .005, Figure 3(b)). A recent report suggests that these so-called myeloid suppressor cells (MSC) exert immunosuppressive effects via secretion of cytokines including a transforming growth factor (TGF)-*β* [21]. Hypothetically, these MSC may derive from ImC accumulating due to disturbed DC differentiation (see Figure 1). After four to five weeks of treatment with AZD2171, MSC frequencies in the cancer patients went down, although not significantly (***P*** = .08, Figure 3(b)). Of note, normalization upon VEGFR TK inhibition of extremely high predose MSC frequencies in two patients (clear outliers of > 2% of PBMCs in Figure 3(b)) was in both cases followed by a minor clinical response.

#### 3.2.3. Myeloid (MDC-1 and MDC-2) and plasmacytoid (PDC) DC subsets

**Figure 4:**
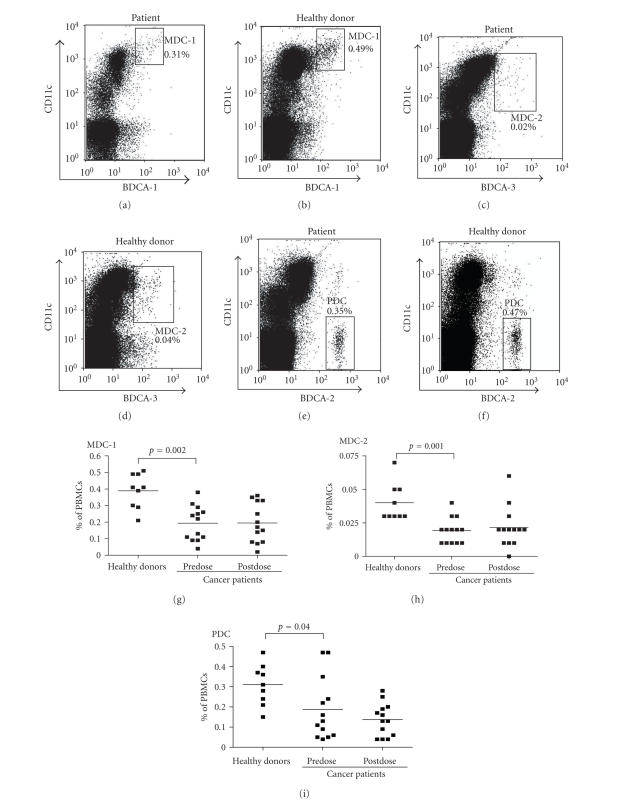
Typical results of peripheral blood DC monitoring based on BDCA marker analysis of a patient with fibrosarcoma (a, c, e) and one healthy donor (b, d, f). Plots for MDC-1 (BDCA-1/CD1c^+^ (a, b)), MDC-2 (BDCA-3/CD141^+^ (c, d)), and PDC (BDCA-2/CD303^+^ (e, f)) are shown. In all plots, peripheral blood DC percentages are of total peripheral blood mononuclear cells and based on the absence of CD14 expression. MDC-1 frequencies in healthy donors and in cancer patients before (predose) and after VEGFR inhibition (postdose) (g), MDC-2 frequencies in healthy donors and in cancer patients before (predose) and after VEGFR inhibition (postdose) (h), and PDC frequencies in healthy donors and in cancer patients before (predose) and after VEGFR inhibition (postdose) (i) are shown. Peripheral blood DC percentages are of total peripheral blood mononuclear cells. In all graphs, individual values and the means are shown.

Typical pretreatment FACS data for MDC-1 (BDCA-1/CD1c^+^), MDC-2 (BDCA-3/CD141^+^), and PDC (BDCA-2/CD303^+^) from one patient are shown in Figure 4, next to comparable data from a healthy donor. Pretreatment MDC subset frequencies in the mononuclear cell population were significantly lower in the blood of cancer patients as compared to healthy donors (MDC-1: patients 0.19±0.10 % [mean ± SD] versus controls 0.39±0.11 %, ***P*** = .002; MDC-2: patients 0.019±0.01 % versus controls 0.04±0.01 %, ***P*** = .001; Figures 4(g), 4(h)). Of note, PDC frequencies in the blood of the cancer patients were also significantly decreased (patients 0.19±0.15 % versus controls 0.31±0.10 %; ***P*** = 0.04; Figure 4(i)). Treatment with AZD2171 did not raise the frequencies of the MDC-1, MDC-2, or PDC subsets in patients with advanced cancer (Figures 4(g)–4(i)).

Absolute numbers of the measured DC subsets per mL of blood were also calculated, and these numbers were not significantly affected by the TKI therapy either (data not shown). Apart from the anecdotal observations for the pMDC and MSC discussed above, no further correlations could be established between the dose of AZD2171 or the tumor response and the measured ImC, PBDC, or MSC frequencies.

Since patients received daily dosing of both AZD2171 and 250 mg gefitinib, we also included four advanced NSCLC patients receiving 250 mg gefitinib monotherapy to distinguish between any effects of VEGFR and EGFR inhibition. Gefitinib monotherapy did not affect the PBDC nor the MSC frequencies in the blood of the studied cancer patients (data not shown), consistent with the observed lack of EGFR expression on these cell populations.

### 3.3. PBDC maturation status before and after VEGFR TK inhibition

To assess the maturation status of the pMDC, MDC, and PDC subsets before and after VEGFR TK inhibition, expression levels of CD86 and/or HLA-DR were determined. Median fluorescence indices for healthy donors and cancer patients are listed in Table 1. No significant differences in CD86 and/or HLA-DR expression levels on the pMDC, MDC, and PDC subsets were found between healthy volunteers (n = 8) and tested cancer patients (n = 13), neither before nor after treatment with AZD2171.

### 3.4. Circulating VEGF levels

To correlate the levels of tumor-derived VEGF to PBDC frequencies, serum levels of VEGF were measured. We measured 2-fold higher levels of serum VEGF in the cancer patients at baseline than previously reported in healthy donors [22]. Interestingly, serum VEGF levels in cancer patients tended to increase after four to five weeks of treatment with AZD2171 and gefitinib with a mean pretreatment VEGF level of 626 pg/ml (range from 114 to 2847 pg/ml) and a mean posttreatment level of 947 pg/ml (range from 245 to 3360 pg/ml; ***P*** = .2). VEGFR TK inhibition is known to up-regulate circulating VEGF levels, which has previously been demonstrated for multiple VEGFR TK inhibitors [23, 24]. In our studies, no correlation was found between serum VEGF levels and ImC, PBDC, or MSC frequencies, neither before nor after treatment. In addition, no correlation was observed between serum VEGF levels and clinical outcome.

## 4. DISCUSSION

Since VEGF has been shown to block DC differentiation and maturation in preclinical models, high levels of VEGF in cancer patients may induce an accumulation of immature and functionally impaired DC contributing to tumor escape from immunosurveillance. As indicated in Figure 1 and based on previous reports [3], we hypothesized that tumor-derived VEGF might exert its inhibitory effect at the stage of immature HLA-DR^−^MDC precursors within the ImC fraction blocking their development into pMDC, while simultaneously skewing their differentiation towards a newly identified population of myeloid CD14^+^HLA-DR^neg/low^ suppressor cells with immunosuppressive traits [21].

We, therefore, evaluated the effect of administration of the VEGFR TKI AZD2171 on PBDC precursor and subset frequencies in advanced cancer patients. It is important to keep in mind that conclusions drawn from this study may be hampered by the applied phase I study design, including multiple dose levels and a heterogeneous patient population. Nevertheless, we found an increased number of immature DC precursors in cancer patients, although this difference did not reach the level of significance. We also identified a significantly increased number of CD14^+^HLA-DR^neg/low^ MSC in the blood of cancer patients as compared to healthy donors (***P*** = .005), which tended to be lower after four or five weeks of VEGFR inhibition. Furthermore, we found that the frequencies of pMDCs, including MDC-1 and MDC-2, were significantly reduced in advanced cancer patients as compared to healthy individuals. In addition, PDC frequencies were significantly reduced in cancer patients compared to healthy donors as previously reported for patients with Kaposi sarcoma or advanced prostate cancer [25, 26]. These results point to a generalized defective DC differentiation, involving multiple DC lineages, across a variety of different tumor types. Thus the effect of advanced tumors on DC differentiation is systemic and results in a profound reduction of mature DC precursors in the circulation and a simultaneous accumulation of immature myeloid DC precursors with a potentially immunosuppressive role (Figure 1), in line with previous reports [3, 4, 6]. After a period of four to five weeks of AZD2171 administration, we did not observe an overall significant increase in pMDC (or indeed, PDC) frequencies, nor did we observe a difference between CD86 and/or HLA-DR expression levels on pMDCs and PBDCs in cancer patients versus healthy donors. This is in contrast to earlier findings by Almand et al. [3], who reported a lower expression of costimulatory molecules on immature myeloid cells.

Might the duration and dosing of AZD2171 administration have been insufficient to effect a reversal of the observed systemic DC differentiation? A period of four or five weeks of AZD2171 administration should be sufficient to affect PBDC frequencies, since frequencies of DC precursors were reported to improve already three to four weeks after tumor resection [3, 5]. Although it remains to be formally proven that AZD2171 actually inhibits the phosphorylation of VEGFR on PBMCs, pharmacokinetic data show that, after multiple daily dosages of 20, 30, or 45 mg of AZD2171, biologically active plasma concentrations are reached, sufficient for sustained VEGFR-1, -2, and -3 inhibition with subsequent effects on clinical parameters, for example, a clear rise in blood pressure [23]. Furthermore, the pharmacokinetics of AZD2171 was not affected by coadministration of gefitinib (van Cruijsen et al., Proceedings of the 41^st^ annual meeting of the American Society of Clinical Oncology, 2005). Rather than by these pharmacodynamic or kinetic considerations, the lack of effect on PBDC frequencies of VEGFR TK inhibition in our study may be explained by the advanced disease state of the participating patients, which likely resulted in a redundancy of DC suppressive factors. Besides VEGF, other cytokines secreted by tumor cells are involved in the inhibition of DC differentiation and maturation, among others are IL-6, IL-10, and M-CSF [27–29]. These other soluble tumor-derived factors may thus overrule the potentially beneficial effect of VEGFR signaling blockade, particularly in late stages of cancer development, which are associated with relatively high systemic levels of these suppressive factors. VEGF may also exert its effect on DC differentiation via another mechanism unlike phosphorylation of VEGFR on DC [30, 31]. A small trial using bevacizumab (a monoclonal antibody binding and neutralizing VEGF) in combination with chemotherapy did report improved DC frequencies after treatment [3]. These results, which are in contrast to our findings using AZD2171, a TKI of VEGFR, might indicate an indirect, TK-independent effect of VEGF on DC differentiation.

Although we did not observe an effect of VEGFR TK inhibition on PBDC precursors and subsets in cancer patients, the frequencies of accumulated MSCs tended to decrease after AZD2171 treatment. This CD14^+^HLA-DR^neg/low^ MSC population may be the human equivalent of CD11b^+^ and Gr-1 myeloid suppressive cells identified in mice [32, 33], and we hypothesized that tumor-derived VEGF might have skewed the hematopoiesis towards an expansion of these myeloid cells with immunosuppressive traits. In mice, this scarce population of immunosuppressive cells could be increased by tumor-derived factors while neutralizing VEGF-antibodies inhibited expansion of this myeloid subset [34]. Reduction of MSC in murine models has been shown to facilitate the rejection of established metastatic disease [35]. It is notable, in this regard, that both the two patients with high pretreatment MSC frequencies, which normalized upon VEGFR TKI administration, had a minor clinical response. One of these also showed a simultaneous increase in pMDC to normal levels. However, due to the nature and size of this Phase I trial, these clinical observations remained anecdotal.

Ideally, phenotypic analyses of MSC and PBDC precursors and subsets should be accompanied by functional assays. Large volumes of blood would have been needed to evaluate the effect of AZD2171 on PBDC function, which made this an unfeasible approach in the current setting. Additional in vitro studies are, therefore, ongoing to assess the effect of VEGFR TK inhibition on DC and MSC differentiation and functionality.

In conclusion, our results indicate that advanced cancer patients harbor increased immature myeloid DC precursor and MSC frequencies, both with potential immunosuppressive effects, as well as reduced MDC and PDC frequencies in their circulation. VEGFR TK inhibition by AZD2171 with antiangiogenic and preliminary anticancer effects did not appear to change any of these DC (precursor) frequencies, although a trend was observed towards reduced MSC frequencies. Our results support the idea that tumor-induced inhibition of DC differentiation is systemic and most likely caused by multiple factors. Clinical approaches to reverse this process should, therefore, encompass systemic blockade of additional tumor-derived immunosuppressive cytokines besides VEGF.

## Figures and Tables

**Table 1:** Expression of CD86 and HLA-DR on PBDC subsets^a^.

PBDC subset	pMDC	MDC-1		MDC-2		PDC	
	HLA-DR	CD86	HLA-DR	CD86	HLA-DR	CD86	HLA-DR
Healthy donors^b^	60.4	18.4	802	5.4	517.1	2.5	295.7
	(7.3–274.8)	(7.2–31.6)	(403.3–1471.6)	(3.0–6.4)	(196.4–1251.4)	(1.3–3.5)	(165.6–469.7)
Cancer patients^c^, predose	63.9	17.5	650.4	10.4	317.5	2.6	253.6
	(19.9–212.4)	(7.7–55.5)	(166.2–1028.1)	(2.3–41.4)	(46.9–854.5)	(1.2–3.3)	(61.8–528.6)
Cancer patients, postdose^d^	51.9	13.1	495.3	7.5	264.2	2.5	251.2
	(26.2–134.1)	(7.9–33.7)	(264.2–842.6)	(2.2–11.0)	(139.6–395.8)	(1.6–4.0)	(123.6–427.4)

^a^Mean and range of median fluorescence index are listed. ^b^Healthy donors, n = 8 ^c^Cancer patients, n = 13 ^d^Postdose, after 4-5 weeks of VEGFR inhibition.
